# Clozapine‐induced slowing in quantitative EEG: Delta–theta amplification and alpha peak shift in TRS patients

**DOI:** 10.1002/pcn5.70186

**Published:** 2025-08-11

**Authors:** Ryo Sawagashira, Shuhei Ishikawa, Atsuhito Toyomaki, Toru Horinouchi, Naoki Hashimoto, Takahiro A. Kato

**Affiliations:** ^1^ Department of Psychiatry, Graduate School of Medicine Hokkaido University Sapporo Japan; ^2^ Health Care Center Hokkaido University Sapporo Japan

Clozapine is the only antipsychotic approved specifically for treatment‐resistant schizophrenia (TRS).^1^ Clozapine is associated with an increased risk of seizures and induces electroencephalogram (EEG) abnormalities in over 50% of patients, although the incidence of clinically evident seizures remains below 5%.^2^ Consequently, prophylactic administration of antiepileptic drugs for asymptomatic EEG changes is of limited clinical value, then routine EEG monitoring is not recommended.^2^ While previous research has primarily focused on epileptiform discharges, recent findings combining machine learning algorithms suggest that clozapine‐related EEG alterations may serve as predictors of treatment response.^3^ Nonetheless, quantitative characterization of slow‐wave activity, particularly within the delta and theta bands, remains insufficient.^4^ Furthermore, most studies rely on qualitative visual assessments rather than objective, frequency‐based methods.^4^ The present study seeks to address these limitations by presenting preliminary data using quantitative EEG analysis.

We conducted a retrospective resting‐state EEG analysis of 15 TRS who initiated clozapine at Hokkaido University Hospital. This study was approved by the Hokkaido University Ethics Committee (approval number: 023–0236). EEGs were obtained at the closest time points before clozapine initiation and after titration to a stable maintenance dose (mean interval: 204 days). The mean age at the time of clozapine initiation was 35.9 years. The mean maintenance dose of clozapine was 308 mg/day. Other demographics was provided in Table S1. Resting‐state EEG was acquired using a standard clinical protocol.^5^ For subsequent offline analysis, EEG data during the eye‐closed condition (obtained from the eye opening/closing test) were selected. The data were segmented into non‐overlapping 1‐s epochs. Epochs were excluded as artifacts if their signal amplitude exceeded ±3 standard deviations from the mean within each channel. Data from eleven electrodes (F3, F4, C3, C4, P3, P4, O1, O2, Fz, Cz, Pz) were included in the analysis. Figure 1a shows representative individual traces (black) and the corresponding mean waveforms before (blue) and after (red) clozapine administration from O1 channel in a representative TRS patient.

**Figure 1:**
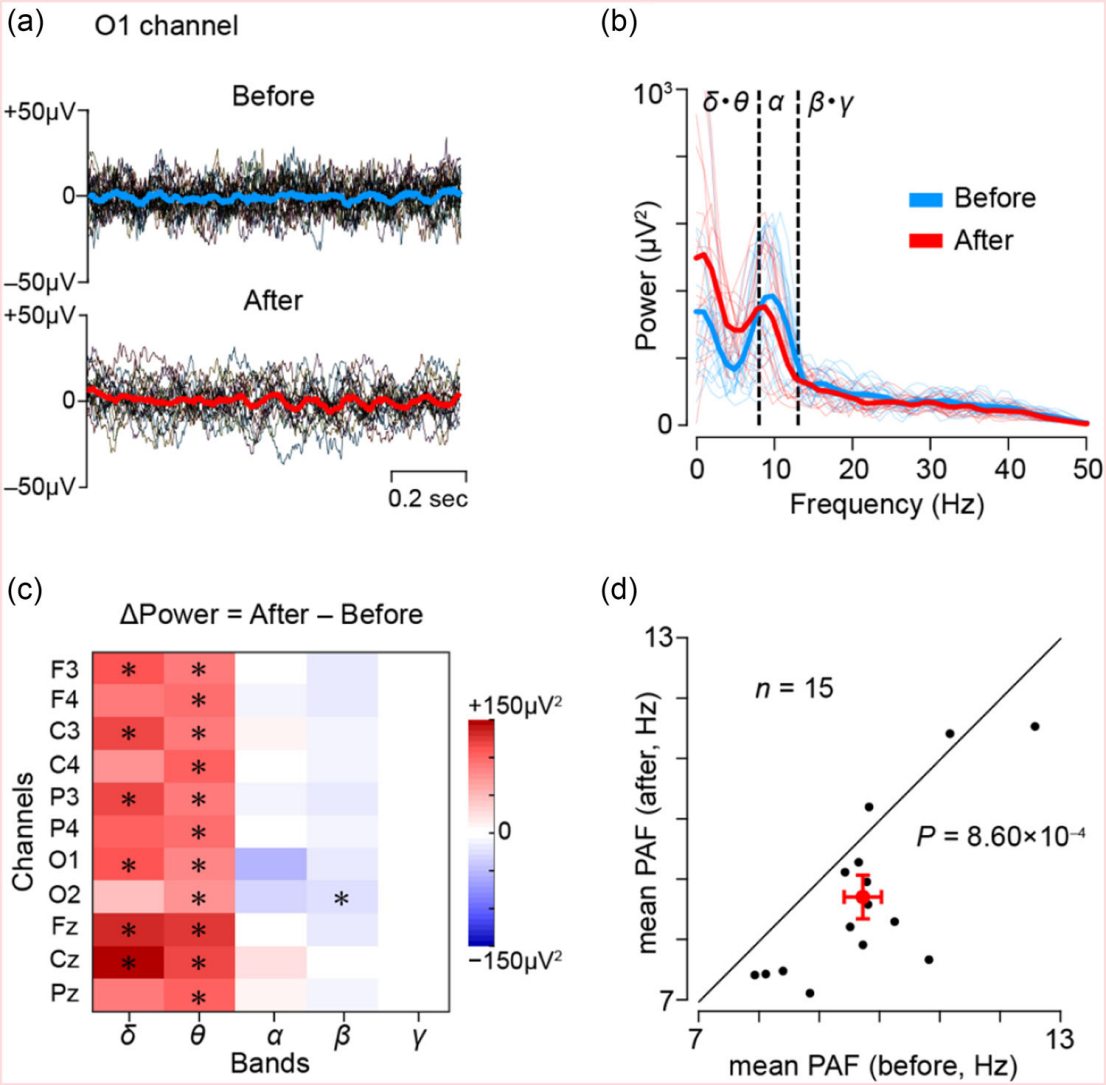
Electroencephalogram (EEG) changes before and after clozapine initiation in patients with treatment‐resistant schizophrenia (TRS). (a) Representative EEG waveforms recorded from the O1 channel in a 41 y.o TRS patient. Thin black lines indicate individual trials (epochs) obtained from eyes‐closed conditions, then segmented. Blue and red lines represent the mean waveforms before and after clozapine initiation, respectively. (b) Power spectral density calculated from the data shown in panel A. Blue and red lines represent the average spectra before and after clozapine initiation, respectively. (c) Heatmap of mean changes in spectral power (post − pre) across eleven channels (F3–Pz) and five frequency bands in 15 patients. Black asterisks indicate significant changes after FDR correction using the Benjamini–Hochberg method (*q* < 0.05). (d) Scatter plot of mean peak alpha frequency (PAF) across eleven channels (F3–Pz) before and after clozapine initiation in 15 patients. Each dot represents one subject. The red dot and error bars indicate the group mean and 95% confidence interval, respectively.

Epochs that passed artifact rejection were subjected to Fast Fourier Transformation to compute spectral power in delta (1–4 Hz), theta (4–8 Hz), alpha (8–13 Hz), beta (13–30 Hz), and gamma (30–100 Hz) bands. Power spectral density values were averaged across all epochs for each subject and channel. Peak alpha frequency (PAF), an established neurophysiological marker associated with cognitive processing and arousal levels, was also analyzed.^6^ PAF was computed as the frequency showing the maximum power within the alpha band for each epoch, and then averaged at the subject level (Figure 1b).

Two robust effects emerged: amplification in delta/theta power, and a downward shift in PAF. Figure 1c displays a heatmap of spectral power changes (post–pre), showing significant increases in delta/theta power in the majority of channels (17 out of 22, FDR‐corrected *p* < 0.05). This pattern remained consistent regardless of lithium carbonate use (see Figure S1). This suggests that clozapine administration caused a widespread pattern of low‐frequency amplification. Furthermore, mean PAF across multiple channels significantly decreased after clozapine initiation (paired *t*‐test, *t*_(14)_ = 4.22, *p* = 8.60 × 10⁻⁴; Figure 1d).

Clozapine is known to strongly antagonize muscarinic M1 receptors, which are critical for generating and modulating cortical oscillations.^7^ Our findings of delta/theta enhancement and alpha slowing are consistent with M1 receptor blockade and reduced cholinergic tone.^8^ These findings are further supported by our previous studies comparing clozapine‐treated schizophrenia spectrum patients with drug‐free individuals.^9^ Taken together, these results suggest that clozapine may induce widespread cortical slowing through cholinergic disruption. These results provide quantitative evidence of clozapine‐induced EEG slowing, particularly in lower‐frequency bands, and suggest that EEG markers such as delta/theta power and PAF may reflect treatment‐related neurophysiological changes. While speculative, these alterations could be associated with either therapeutic mechanisms or potential adverse effects; however, no direct clinical or cognitive measures were assessed in this study. A recent meta‐analysis^10^ reported that clozapine treatment was associated with cognitive impairment across multiple domains. Although the present study did not include cognitive assessments, the observed EEG slowing has previously been linked to cognitive dysfunction in the literature, suggesting a potential association that merits further investigation. Specifically, a reduction in PAF has previously been linked to cognitive decline and sedation in both psychiatric and non‐psychiatric populations.^6^ Whether such changes represent beneficial mechanisms or adverse outcomes remains unclear. Future studies should clarify these possibilities by integrating more frequent EEG monitoring with standardized cognitive testing, clinical symptomatology, and serum levels of clozapine and its active metabolite, N‐desmethylclozapine.

## AUTHOR CONTRIBUTIONS

Ryo Sawagashira conceptualized the study, analyzed the data, and drafted and revised the manuscript. Shuhei Ishikawa, Atsuhito Toyomaki, and Toru Horinouch supervised data interpretation and manuscript editing. Naoki Hashimoto and Takahiro A. Kato contributed to the critical revision of the manuscript. All authors reviewed and approved the final version of the manuscript.

## CONFLICT OF INTEREST STATEMENT

The authors declare no conflict of interest.

## ETHICS APPROVAL STATEMENT

This study was approved by the Ethics Committee of Hokkaido University Hospital (approval number: 023–0236) and conducted in accordance with the Declaration of Helsinki.

## PATIENT CONSENT STATEMENT

In accordance with institutional guidelines for retrospective studies, written informed consent was waived, and information about the study was disclosed publicly to allow patients the opportunity to opt out.

## CLINICAL TRIAL REGISTRATION

N/A.

## Supporting information

Supplementary Table 1. Demographic characteristics.

Supplementary Figure 1. EEG changes before and after clozapine initiation in patients with TRS with and without lithium therapy.

## Data Availability

The data sets generated and analyzed during the current study are not publicly available due to ethical restrictions, but de‐identified data may be available from the corresponding author on reasonable request.
