# Supplementary material for: Clozapine‐induced slowing in quantitative EEG: Delta–theta amplification and alpha peak shift in TRS patients
Source: PCN Rep. 2025 Aug 11;4(3):e70186. doi: 10.1002/pcn5.70186 (PMC12339658; doi:10.1002/pcn5.70186)
Supplement: Supplementary file 1 — Supplementary Table 1. Demographic characteristics. [file PCN5-4-e70186-s001.docx]

**Supplementary Table 1. Demographic characteristics**

|  |  |  |  |  | **Post** | | | **Pre** | |
| --- | --- | --- | --- | --- | --- | --- | --- | --- | --- |
| **Sex** | **Age** | **Education (year)** | **Duration of illness (year)** | **EEG interval (day)** | **CLOZ (mg)** | **Li (mg)** | **BZPs (mg)** | **BZPs (mg)** | **Antipsychotics (mg)** |
| F | 26 | 14 | 9 | 92 | 225 | 600 | 0 | 0 | BRX 2 |
| M | 35 | 18 | 12 | 149 | 200 | 200 | 2.5 | 7.5 | ARP 24 |
| F | 35 | 14 | 17 | 99 | 75 | 0 | 0 | 10 | PAL 9 |
| F | 21 | 12 | 1 | 479 | 200 | 0 | 20 | 0 | OLZ 5 |
| F | 49 | 12 | 29 | 69 | 175 | 0 | 0 | 15 | HAL 8 + CP 200 + LP 50 |
| M | 30 | 12 | 2 | 40 | 200 | 0 | 0 | 5 | OLZ 20 |
| F | 29 | 12 | 9 | 243 | 350 | 400 | 0 | 8.3 | BLO 24 + PER 24 |
| M | 57 | 13 | 5 | 100 | 225 | 400 | 5 | 15 | RIS 1 + QTP 300 |
| M | 29 | 12 | 14 | 538 | 600 | 0 | 25 | 0 | PER 16 + LP 5 |
| M | 41 | 16 | 12 | 647 | 175 | 600 | 5 | 0 | QTP 450 + BRO 18 |
| M | 31 | 12 | 10 | 120 | 500 | 0 | 0 | 2.0 | HAL 6 |
| F | 34 | 14 | 12 | 56 | 150 | 600 | 0 | 35 | HAL 6 + CP 50 |
| F | 39 | 12 | 11 | 86 | 500 | 0 | 25 | 46 | QTP 600 + BLO 24 |
| F | 39 | 12 | 23 | 166 | 500 | 400 | 20 | 5 | RIS 12 + LP 50 |
| M | 44 | 12 | 9 | 175 | 550 | 0 | 0 | 25 | QTP 300 |
| CLOZ: clozapine; Li: lithium carbonate; BZPs: benzodiazepines (converted to diazepam-equivalent dose); BRX: brexpiprazole; ARP: aripiprazole;  HAL: haloperidol; CP: chlorpromazine; LP: levomepromazine; OLZ: olanzapine; BLO: blonanserin; PAL: paliperidone; PER: perospirone; | | | | | | | | | |
| RIS: risperidone; QTP: quetiapine; BRO: bromperidole | | | | | | | | | |
