# Supplementary material for: Clozapine‐induced slowing in quantitative EEG: Delta–theta amplification and alpha peak shift in TRS patients
Source: PCN Rep. 2025 Aug 11;4(3):e70186. doi: 10.1002/pcn5.70186 (PMC12339658; doi:10.1002/pcn5.70186)
Supplement: Supplementary file 2 — Supplementary Figure 1. EEG changes before and after clozapine initiation in patients with TRS with and without lithium therapy. [file PCN5-4-e70186-s002.docx]

**
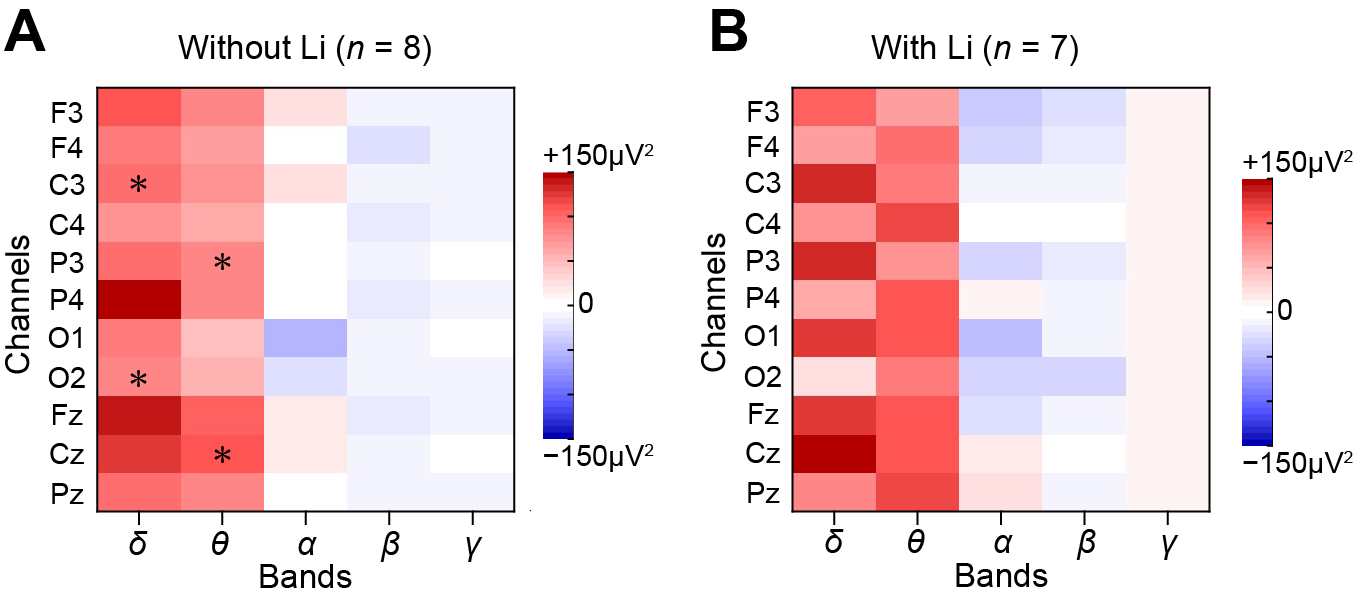
**

**Supplementary Figure 1. EEG changes before and after clozapine initiation in patients with TRS with and without lithium therapy.**
Heatmap of mean changes in spectral power (post − pre) across eleven channels (F3–Pz) and five frequency bands in 8 patients without lithium carbonate **(A)** and 7 patients with lithium carbonate therapy **(B)**. Black asterisks indicate statistically significant changes after false discovery rate (FDR) correction (P < 0.05).
